# Superconductivity in two-dimensional ferromagnetic MnB

**DOI:** 10.1038/s41598-017-17235-y

**Published:** 2017-12-06

**Authors:** M. Umar Farooq, Arqum Hashmi, Imran Khan, Jisang Hong

**Affiliations:** 1 Department of Physics, Pukyong National University, Busan, 608-737 Korea; 2 Center for Computational Sciences, University of Tsukuba, Tsukuba, 305-8577 Japan

## Abstract

Using the universal structure predictor algorithm, we proposed that two-dimensional MnB structures with p4mmm (α-MnB) and pmma (β-MnB) symmetries could be synthesized. This finding was verified by calculating the dynamical stability, molecular dynamics, and mechanical properties. The α-MnB had an in-plane stiffness Y_*x*_ (=Y_*y*_) around 100 N/m while the β-Mn displayed an asymmetric mechanical stiffness of Y_*x*_ = 186 N/m and Y_*y*_ = 139 N/m. Both systems displayed a ferromagnetic ground state with metallic band structures. The calculated magnetic moments were 2.14 and 2.34 µB per Mn-B pair in the α-MnB and β-MnB. Furthermore, we investigated the potential superconductivity. In the α-MnB, we found the unique feature of Kohn anomaly at q~2k_F_ in the diagonal direction of the Brillouin zone. The β-MnB phonon spectra showed a valley of degenerated localized softening vibration modes at the edge of the Brillouin zone. The ZA and LA phonon branches in this valley induced the largest contribution to electron-phonon coupling strength. The calculated total electron-phonon coupling parameters were 1.20 and 0.89 in α-MnB and β-MnB systems. Overall, we predict that the α-MnB and β-MnB systems can display 2D ferromagnetic superconducting states with the estimated critical temperatures of Tc ≈ 10−13 K.

## Introduction

Discovery of two-dimensional (2D) graphene is unparalleled to any other findings in the field of condensed matter physics. The alluring physical properties of graphene ignite the surge for the quest of other 2D materials. For instance, the experimental realization of graphene has prompted the idea of the development of other 2D materials from their bulk three-dimensional (3D) allotropes. So far, various types of 2D materials such as phosphorene, silicene, germanene, 2D boron nitrite and transition-metal dichalcogenides have been introduced^1–3^. On the other hand, extensive research efforts have been performed to fabricate carbon and nitrogen based polymer like 2D materials such as C_2_N, g-C_4_N_3_ and C_3_N_4_ and various physical properties are investigated^4–7^. More recently, 2D transition metal oxides nanosheets was also experimentally demonstrated by a self-assembly method^8^. On the theoretical side, the reverse density functional approach is emerging as a successful method for designing of utterly new materials as well as for finding different structural and stoichiometric relatives of the pre-existing materials. In this technique, the universal structure predictor algorithm is used to discover materials and this technique is a powerful tool to streamline the experimental efforts in the synthesis of new 2D materials for particular physical properties for the hand-picked physical, structural or stoichiometric properties^9^. For instance, it was very successful in theoretical predictions of 2D borophene structure and helping experimentalist in fabrication^10,11^.

Superconductivity has long been the cutting-edge problem in the condensed matter physics. Particularly, the discovery of magnetic superconductors has raised an intriguing issue regarding the origin of superconductivity^12,13^. Recently, the superconductivity with the reduced dimensionality is attracting great research interest. For instance, the potential superconductivity in 2D phosphorene and borophene was reported although their bulk allotropes did not show any indication of superconductivity^14–16^. Besides, most of previously reported 2D superconducting materials are non-magnetic materials. Thus, the study on 2D magnetic superconductivity will be an interesting subject. Very recently, it was shown that the monolayer FeSe could show superconductivity with an antiferromagnetic (AFM) state^12,17^. In addition, Zhu *et al*. reported a ferromagnetic (FM) superconductivity in the 2D NbSe_2_ with surface molecular adsorption^18^. However, the molecule adsorption creates the localized magnetic state and this localized feature limits its uses for spintronic applications. Despite the numerous studies on 2D materials, only a few systems display an intrinsic FM ground state. Due to this, the study on 2D FM superconductivity is still in early stage. Thus, it will be an intriguing finding if we can suggest a superconducting property with an intrinsic ferromagnetic material. Recently, it was found that the bulk MnB can display a room temperature FM ground state^19^. This room temperature ferromagnetism will be an important feature for spintronics applications. Nonetheless, so far, no studies have been performed whether the 2D MnB structure can be formed with FM ground state. In this report, we will show that the 2D MnB stoichiometric crystal structures can be realized with a FM ground state. Moreover, we aim to propose that the FM superconductivity can be found in this MnB structure.

## Results

**Figure 1:**
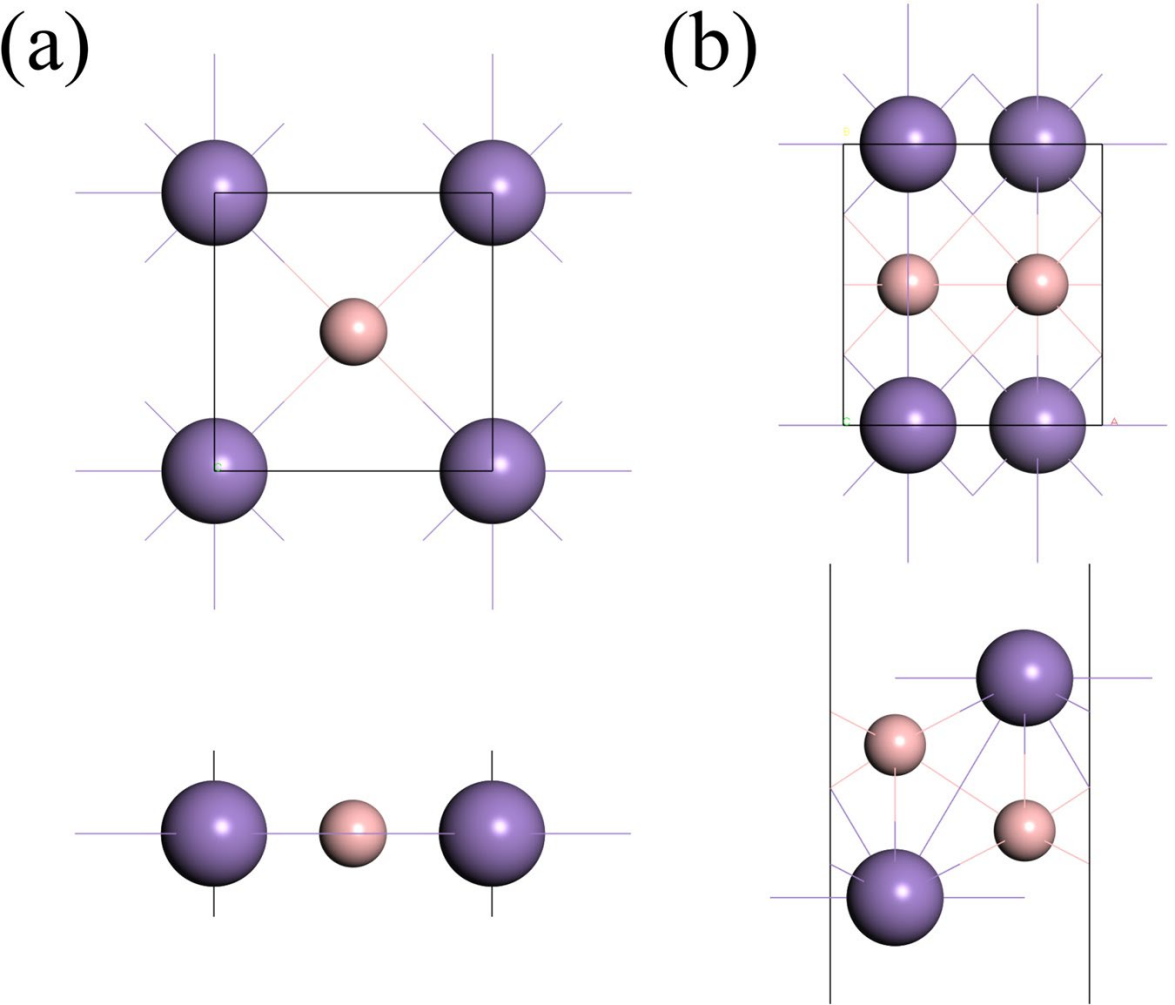
Crystal structure of single unit-cells with top and side views, pink balls representing boron atoms and violet are denoting Mn atoms (**a**) for α-MnB and (**b**) for β-MnB.

We employed the ab-initio global energy minima search to find the most stable structures for one and two MnB pairs. The details can be found in the computational details section. Using this method, we found that the lowest energy structure had a p4mmm symmetry with lattice constants of a = b = 2.875 Å and another structure has a pmma symmetry with lattice constants of a = 2.933 Å and b = 3.180 Å. With these structures, we investigated the dynamical stability by calculating the phonon dispersion curve. No imaginary frequencies were found in Brillouin zones (BZ) of these two energetically stable MnB structures. Figure 1(a,b) shows the top and the side views of p4mmm–MnB (namely α-MnB) and pmma–MnB (namely β-MnB). As one can clearly see, the α-MnB has a simple square lattice and it has two atoms in a unit cell. According to best of our knowledge, no previous report is available for the possibility of 2D material with a square symmetry. Most of recently found 2D systems have very asymmetric behaviors in various physical properties along zigzag and armchair directions^20,21^. Hence, the square system with p4mmm structure may give an ideal directional uniformity in many physical properties. On the other hand, the β-MnB has a rectangular lattice structure with alternate chains of Mn and B atoms and it has four atoms in a unit cell.

**Figure 2:**
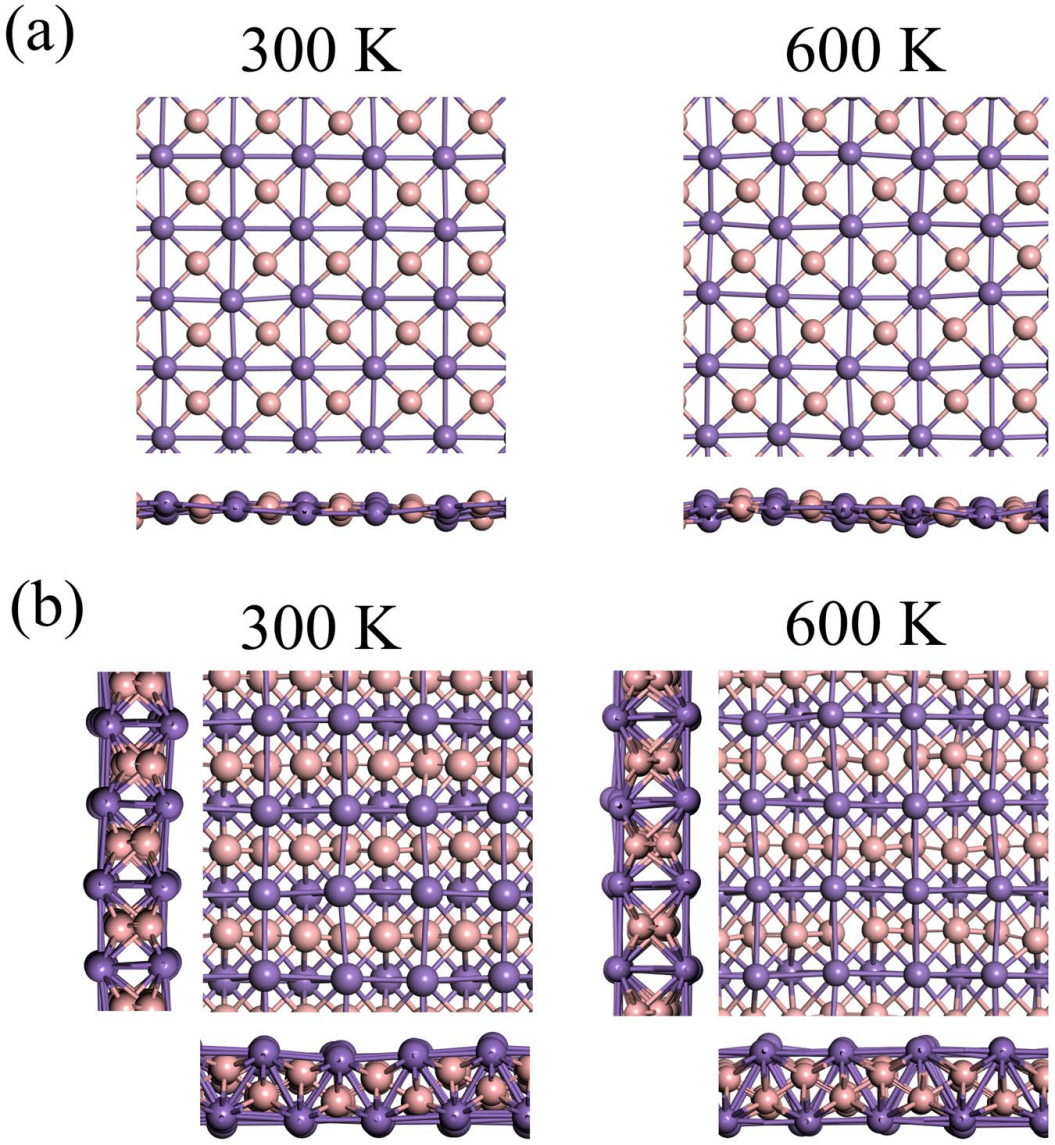
Snapshots of MD at 5 ps with top and side views at 300 K and 600 K for (**a**) α-MnB and (**b**) β-MnB.

Despite the prove of the dynamical stability by phonon dispersions in both α-MnB and β-MnB, it is an important question to raise whether these allotropes can be achievable in realistic conditions or they are completely hypothetical. To answer this question, we calculated the cohesive energy (Ecoh) of 2D MnB structures and compared with the well-known MnB bulk and pre-existing 2D systems such as graphene, silicene, or germanene by using the relation E_coh_ = (xE_Mn_ + xE_B_ − E_tot_)/2x. Here, E_Mn_, E_B_ and E_tot_ are the total energies of a single Mn, single B atom, and the total energy of MnB system. The x indicates the total number of Mn or B atoms in one unit-cell. The cohesive energies of pre-existing 2D materials such as graphene, silicene, and germanene are 7.85, 3.98, and 3.26 eV per atom^22^. In our α-MnB and β-MnB, we obtained the cohesive energies of 4.493 and 4.927 eV per atom and these are comparable to the values found in the pre-existing 2D materials. The calculated cohesive energy of bulk MnB with Pnma symmetry is 5.6 eV which is slightly higher but in the same energy range. These results suggest that both α-MnB and β-MnB monolayer systems can be fabricated in suitable experimental situations. We also evaluated the thermal stability by ab-initio molecular dynamics (MD) simulations at finite temperatures with 5 × 5 and 4 × 4 supercells for α-MnB and β-MnB. Figure 2(a,b) shows the snapshots taken at 5 ps. We found no substantial distortion in their structures even at 600 K. Note that the α-MnB showed a small out-of-plain buckling at 600 K and this is understandable because it has one atomic thick layer. In contrast, the β-MnB consisted of four sub-layer structure and no sizable buckling was found even at 600 K. We also investigated the mechanical stability by calculating the in-plane Young’s modulus Y_S_ (or in-plane stiffness). Due to the square lattice structure in the α-MnB, the in-plane stiffness along x- and y-directions were the same (Y_X_ = Y_Y_) and the calculated in-plane stiffness was Y_X_ = 100 N/m. For the β-MnB, we obtained Y_X_ = 186 N/m and Y_Y_ = 139 N/m. The higher value of stiffness in β-MnB originates from the increased number of bonds. For comparison, we also calculated the in-plane stiffness of monolayer graphene and phosphorene. We obtained 343 N/m for the graphene while the phosphorene had in-plane stiffness values of 93 and 25 N/m for zigzag and armchair directions. Both α-MnB and β-Mn had smaller in-plane stiffness than the one in graphene but higher than that found in the phosphorene. Furthermore, these values are significantly higher than the reported results for a free-standing 2D Ag layer (31 N/m), germaneness (42 N/m), and silicene (61 N/m)^23^. The calculated dynamical stability and mechanical properties suggest that the 2D of MnB system could be synthesized by appropriate experimental techniques on a suitable substrate and these allotropes can exist at ambient conditions.

**Figure 3:**
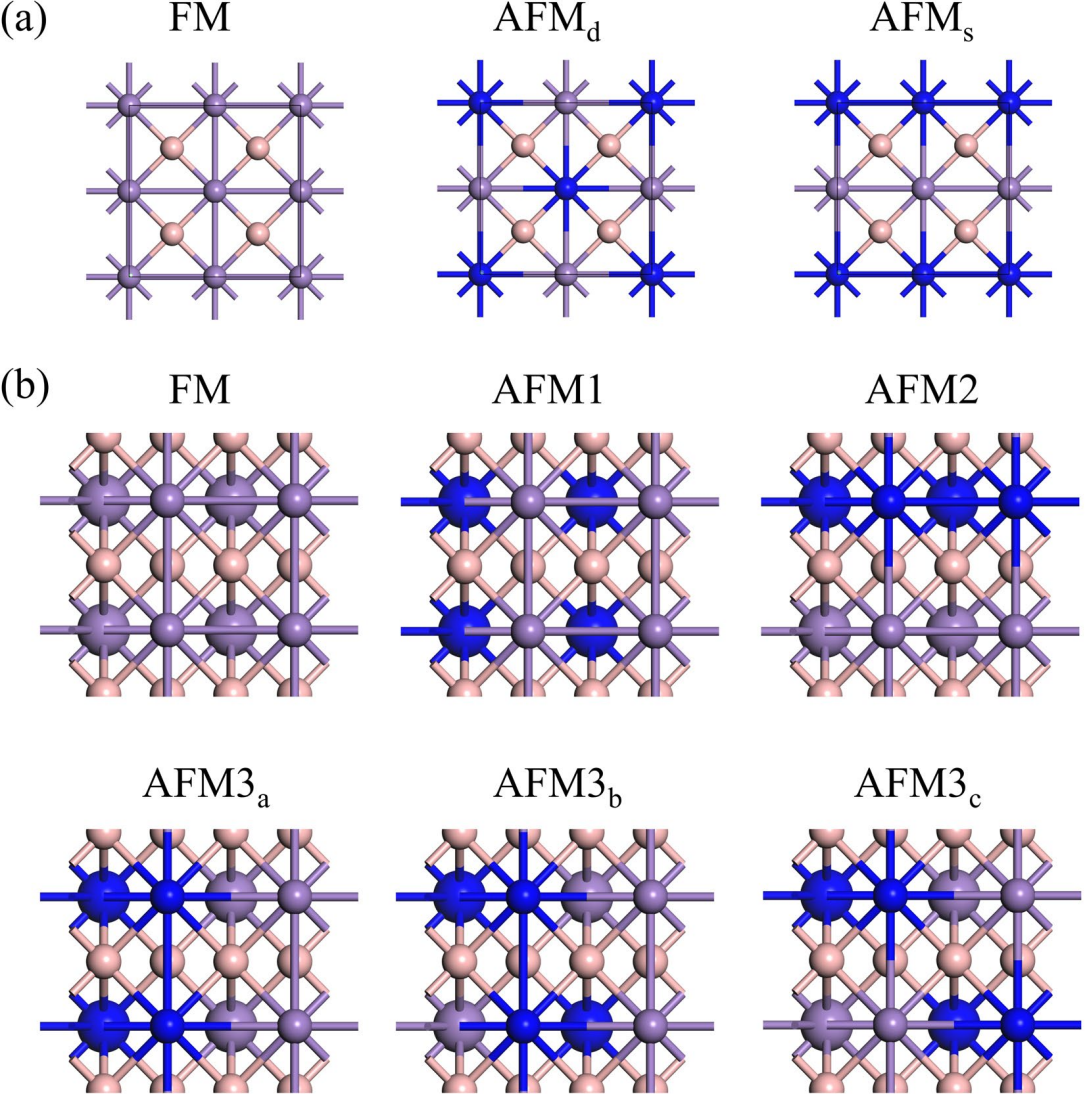
Schematics of possible spin configurations in 2 × 2 cell for (**a**) α-MnB (**b**) β-MnB. Pink balls representing boron atoms, violet and blue balls are denoting alternate spins on Mn atoms. Smaller and larger balls represent the upper and the lower layer Mn atoms.

**Figure 4:**
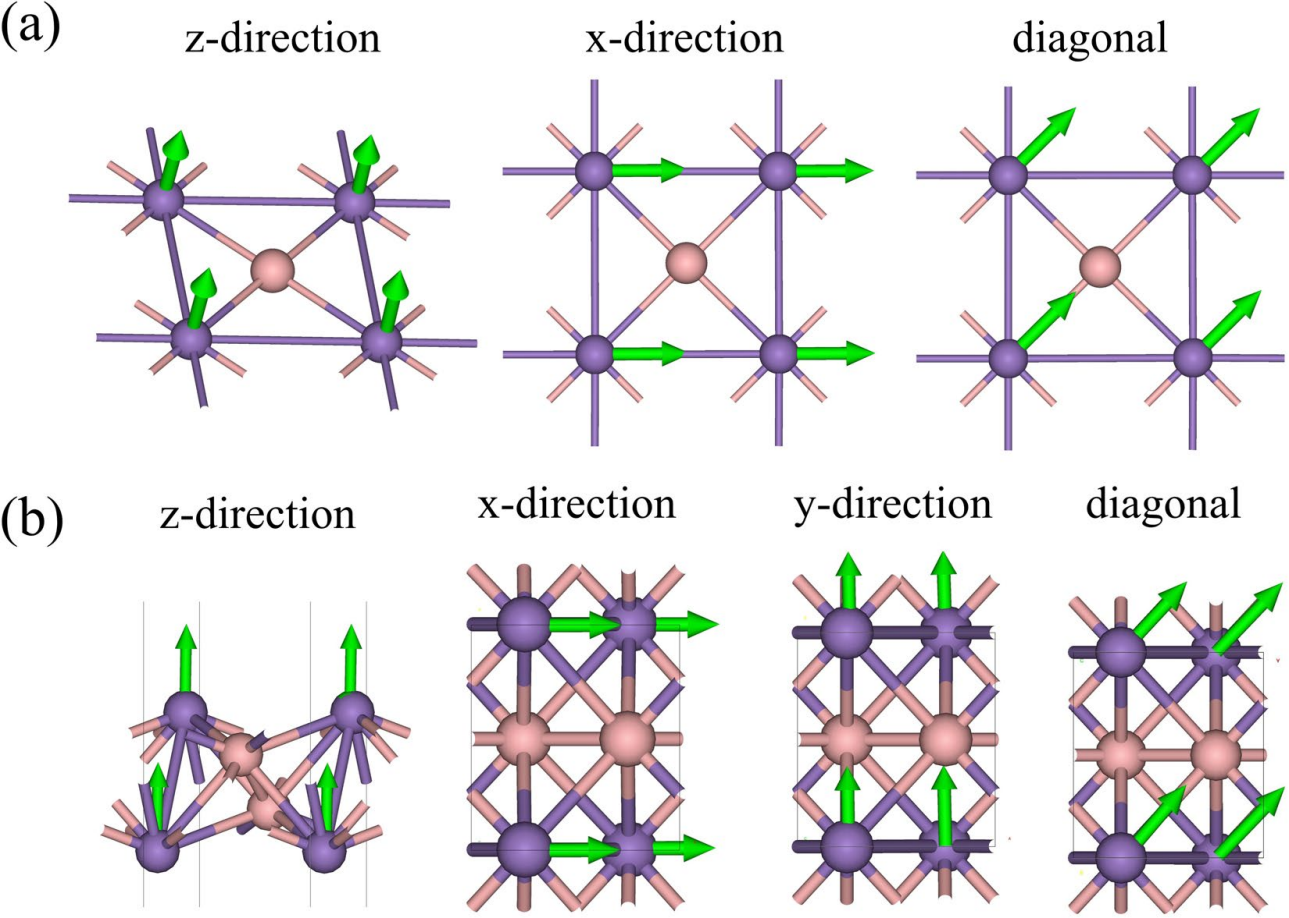
Possible magnetization directions are indicated by the green arrows (**a**) for α-MnB (**b**) for β-MnB. Pink and violet balls represent B and Mn atoms.

We now explore the magnetic state. Due to the square lattice structure, the α-MnB can have three distinct spin configurations and they are displayed in Fig. 3(a). Table 1 shows the calculated total energy difference per Mn-B pair. The total energy in FM state is set to zero as a reference value. We found a FM ground state with a total magnetic moment of 2.14 µB per unit-cell (per Mn-B pair). The Mn had a magnetic moment of 2.3 µB while the B had an opposite magnetic moment of −0.23 µB within a Wigner-Seitz radius. In the β-MnB, two Mn atoms exist in a unit cell; one in the upper layer and the other one in the lower layer and it has six distinct spin configurations as displayed in Fig. 3(b). Table 2 shows the calculated total energy difference per Mn-B pair. The β-MnB had also a FM ground state with a total magnetic moment of 4.69 µB per unit-cell. In the β-MnB, two pairs of Mn-B exist in a unit cell. Thus, the magnetic moment per Mn-B pairs is 2.34 µB. Each Mn atom has a 2.31 µB and each B have a magnetic moment of −0.13 µB within the Wigner–Seitz radius. Taking the magnetic ground state, we calculated the magnetization direction by using the total energy method. To this end, we used non-collinear magnetic configuration including spin-orbit coupling (SOC) with k-mesh grid of 51 × 51 × 1 points. Figure 4(a,b) shows the possible magnetization directions for α-MnB and β-MnB. The square symmetry of α-MnB allows only two distinct in-plane magnetizations; along x-axis or diagonal direction and one out-of-plane directions (z-direction) as shown in Fig. 4(a). However, the β-MnB can have three different in-planes and one out-of-plane direction as in the Fig. 4(b). Table 3 shows the calculated results. The energy for out-of-plane (z) magnetic direction is set to zero as reference. We found that both α-MnB and β-MnB systems had out-of-plane magnetizations.

**Table 1:** Energy difference (in meV) between different spin configurations per Mn-B pair in α-MnB.

System	FM	AFM_d_	AFM_s_
ΔE (meV)/MnB	0.00	129.09	129.09

**Table 2:** Energy difference (in meV) between different spin configurations per Mn-B pair in β-MnB.

System	FM	AFM1	AFM2	AFM3_a_	AFM3_b_	AFM3_c_
ΔE (meV)/ MnB	0.00	151.27	76.06	50.43	36.53	72.72

**Table 3:** Energy difference (in µeV) between different magnetization directions for α-MnB and β –MnB.

System/Directions	out-of-plane z	in-plane x	in-plane y	in-plane diagonal
α-MnB	0.00	—	583.12	583.25
β-MnB	0.00	812.22	370.34	590.86

**Figure 5:**
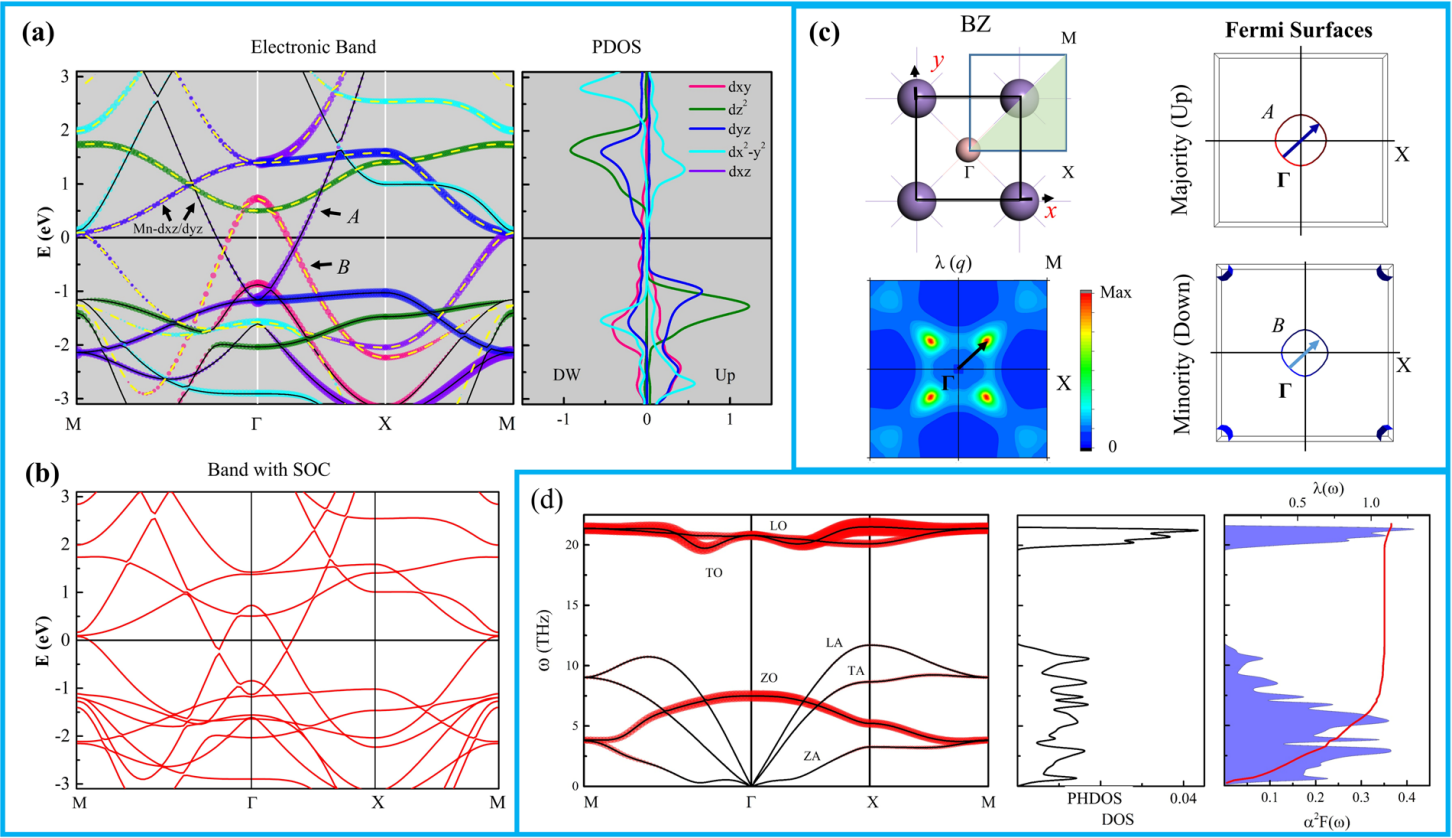
Calculated (**a**) band structure without SOC and PDOS of Mn atoms. The solid black and yellow dashed lines indicate the majority and minority spins (**b**) band structure with SOC (**c**) BZ, Fermi-surfaces for up and down spins, and λ (**q**) in the BZ. The arrows represent 2k_F_ involved in the nesting (**d**) phonon-band with phonon linewidth γ (q, ω) projection where width of red line represents the strength, PHDOS, Eliashberg function α^2^F(ω), and λ (ω). The ZA, TA, LA represent out-of-plane, in-plane transverse and in-plane longitudinal acoustic modes. The ZO, TO, and LO represent out-of-plane, in-plane transverse, and in-plane longitudinal optical modes.

Now, we discuss the electronic structure, electron-phonon coupling (EPC) in relation to the electronic structure and vibrational spectra. Using these properties, we investigate the superconducting features of these materials. Figure 5(a) shows the band structure of α-MnB without SOC and also the partial density of states (PDOS) of Mn. In Fig. 5(b), we present the band structure of α-MnB with SOC. We found that the band structure was not significantly modified even after SOC effect because the SOC strengths of 2p boron and 3d manganese are rather weak. The α-MnB has a metallic band structure and the metallic character is dominated by the Mn-*d* orbitals. The main contribution to the magnetic moment is originated from the Mn-dz^2^ and completely hybridized *d*yz/dxz sates. Near the Γ- point, the Mn*-dxz*/Mn*-dyz* hybridized states in the majority spin band (denoted by *A*) and the in-plane Mn*-dxy* orbital in the minority spin band (denoted by *B*) are crossing the Fermi level. These bands, due to their quadratic dispersions, can form an ideal pair of electron-hole bands with Fermi-surface nesting. Near the M-point, the Mn*-dxz*/Mn*-dyz* hybridized states in the minority spin band crossed the Fermi level. Overall, the bands around Fermi-level are highly dispersive so that the density of state at the Fermi level (N (ε_F_)) is quite weak and this feature is favorable for strong effective momentum-frequency dependent EPC parameter λ(q, ω) because the λ(q, ω) is proportional to the 1/ N (ε_F_). In Fig. 5(c), we show the Brillouin zone (BZ), Fermi surfaces and the frequency integrated effective momentum EPC parameter λ(q) in the BZ. The nesting features of the Fermi surfaces belonging to the majority (*A*) and the minority (*B*) bands are observed along the Γ–M direction. The Fermi nesting could be identified by a very high concentration of λ (q) in a small oval shaped spot elongated in Γ–M direction at q~2k_F_ where q = (0.15, 0.15) in a unit of (2π/a). To describe the dispersion aspect of electron-phonon coupling, we present the phonon dispersion band with phonon line-width projection (γ_qv_), phonon density of states (PHDOS), Eliashberg function (α^2^F(ω)), and q-integrated effective frequency dependent EPC parameter λ (ω) in Fig. 5(d). Since the α-MnB has two atoms in a unit-cell, six phonon frequency modes can exist; three acoustic and three optical phonon modes as labeled in Fig. 5(d). Through the comparison between the phonon-band, PHDOS and Eliashberg function α^2^F(ω), it is clear that the main contribution to the total EPC parameter λ originates from the low-lying out-of-plane optical and acoustic mode. We observed a softening of the ZA mode at approximately q~2k_F_ along the Γ - M and the largest contribution to the integrated effective momentum EPC parameter λ(q) appeared. This feature can be interpreted as Kohn anomaly, a typical behavior in a low- dimensional material^14,15,24^. We also found degenerated ZA and ZO modes near the M point and this feature also induced a considerable EPC. Using this information, we obtained a total EPC parameter λ of 1.2. Since the critical temperature (T_C_) is the most important quantity in the superconductivity, we estimated the T_C_ using the modified McMillan equation, ${T}_{C}=\frac{{\omega }_{log}}{1.2}{\exp }[\frac{-1.04(1+{\rm{\lambda }})}{{\rm{\lambda }}(1-0.62{\mu }^{\ast })-{\mu }^{\ast }}]$ where the ${\omega }_{log}=[\frac{2}{{\rm{\lambda }}}{\int }^{}\frac{d\omega }{\omega }{\alpha }^{2}\,F(\omega )\mathrm{log}\,\omega ]$ and μ* is a parameter to accommodate the effective Coulomb repulsion, typically taken ∼0.1. We obtained the critical temperature of ${T}_{c}^{{\mu }^{\ast }=0.1}\sim 12K\,{\rm{with}}\,{\mu }^{\ast }=0.1$ and λ = 1.2.

**Figure 6:**
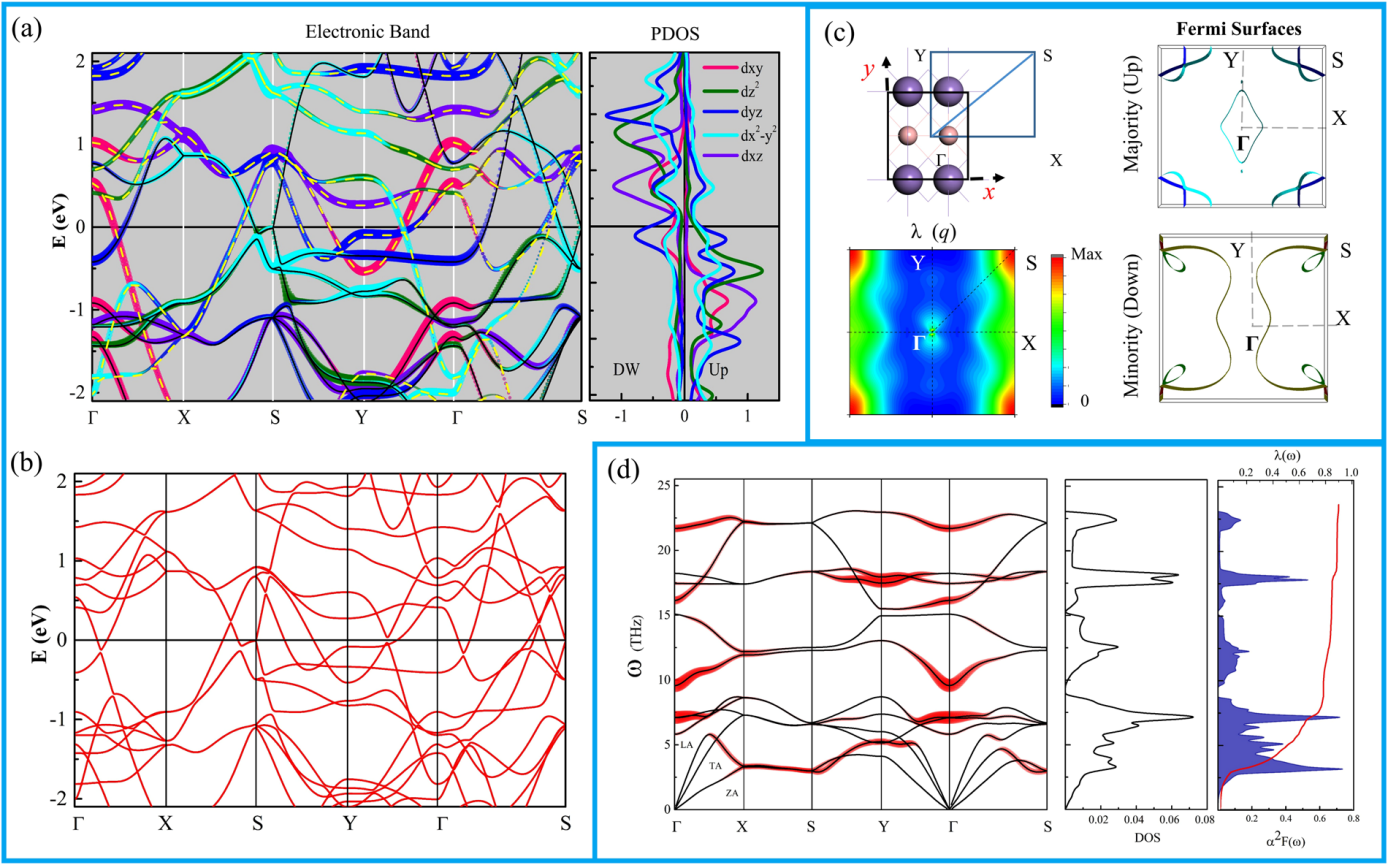
Calculated (**a**) band structure and PDOS of Mn atoms without SOC. The solid black and yellow dashed lines indicate the majority and minority spins (**b**) band structure with SOC (**c**) BZ, Fermi-surfaces for up and down spins, and λ (q) in the BZ (**d**) phonon-band with phonon linewidth γ (q, ω) projection where width of red line represents the strength, PHDOS, Eliashberg function α^2^F(ω), and λ (ω). The ZA, TA, LA represent out-of-plane, in-plane transverse, and in-plane longitudinal acoustic modes.

Figure 6(a) shows the electronic projected band structure of the β-MnB and PDOS of Mn without SOC while Fig. 5(b) is the band structure with SOC. Once again, we found no significant change in the band structure even with SOC effect. The β-MnB also displayed a typical metallic character dominated by the Mn-*d* orbitals. We found two highly dispersive majority and minority spin bands crossing the Fermi level along the Γ - X direction. The majority spin band is dominated by Mn*-d*_yz_ states while the minority spin band is dominated by in-plane orbitals Mn*-d*_*xy*_. Interestingly, the majority spin band dominated by d_x_^2^_−y_^2^ orbital displays Dirac-cone like band dispersions in the S-Y and S-Γ directions. Contrary to the α-MnB case, we observe weak dispersions along the Y - Γ direction around the Fermi level and these states contribute to relatively high DOS near the Fermi level in the β-MnB. This high DOS will contribute to suppressing the λ (q, ω). Figure 6(c) shows the Fermi-surface for both majority, minority spin state, and the λ (q). Unlike the α-MnB, we found relatively complex shapes and the nesting feature was less apparent. Nonetheless, we found a strong λ (q) along the X-S direction and the largest value was observed at S point. Note that relatively weak but noticeable contribution was also observed near the Γ point along the Γ-Y direction. Figure 6(d) shows the phonon-band with phonon linewidth γ(q, ω) projection, phonon density of states(PHDOS), Eliashberg function α^2^F(ω), and effective frequency dependent EPC parameter λ (ω). Since the β-MnB structure has four atoms in a unit-cell, twelve phonon frequency modes are present. The phonon modes are more dispersive along the Γ-X and S-Y directions than along the Γ- Y and X-S directions. This result implies that the β-MnB has a relatively stronger dynamical stability along the x-direction. As discussed earlier, the calculated in-plane stiffness also indicated that the β-MnB had relatively strong mechanical stability along the x-direction. Like in the electronic band dispersion along the X-S direction, most of the phonon modes were also degenerated and made a valley of localized vibration modes. From the Eliashberg function α^2^F(ω) plot, we found that the low-frequency phonon modes strongly contributed to the EPC parameter, especially by acoustic modes. From the phonon band dispersion, we can see the softening and degeneration of ZA and LA branches in the valley of localized modes. Here, this softening of acoustic modes induced the largest contribution to the EPC parameter, particular from S point. Overall, in the β-MnB, we obtained the total EPC parameter λ of 0.89 and the critical temperature of $\,{T}_{c}^{{\mu }^{\ast }=0.1}\sim \,13.5K$.

## Discussion

Through the universal structure optimization process, we obtained two types 2D MnB structures which had p4mmm (α-MnB) and pmma (β-MnB) symmetries and the dynamical stability was confirmed by phonon dispersion calculation. Both α-MnB and β-MnB monolayers had cohesive energies comparable to other 2D materials like graphene, silicene, and germanene. We also performed the MD simulation and found that the systems could be stable at least even up to 600 K. The α-MnB had an in-plane stiffness Y_*x*_ ( = Y_*y*_) around 100 N/m. On the other hand, due to the pmma crystal symmetry, the β-Mn structure displayed an asymmetric mechanical in-plane stiffness of Y_*x*_ = 186 N/m and Y_*y*_ = 139 N/m. Since these values are smaller than that in the graphene but comparable or higher than the value observed in the phosphorene. All these results suggest that the 2D allotropes of MnB can be realized by an appropriate experimental approach. We found a FM ground state in both systems with metallic band structures. In the α-MnB, the Fermi surface nesting was clearly observed and this feature induced a strong EPC. Furthermore, we observed the Kohn anomaly along the diagonal direction of BZ, at q~2k_f_. In the β-MnB, we found the valley of localized softening vibration modes along the X-S direction. The strong contribution to the EPC originated from the ZA and LA phonon branches in this valley. Both 2D allotropes α-MnB and β-MnB showed strong electron-phonon coupling parameters with values 1.20 and 0.89 respectively. Overall, we predict that both α-MnB and β-MnB structures can show potential 2D FM superconductivity with the estimated critical temperature of Tc ≈ 10−13 K.

### Numerical Method

Ab-initio global minima search was performed with a universal structure predictor algorithm^25,26^. In these calculations, 30 initial structures were randomly produced within all 17 planar group symmetries. Theses randomly selected single layer was allowed to have even a buckled geometry. All produced structures were relaxed by using Spanish Initiative for Electronic Simulations with Thousands of Atoms (SIESTA) code with four distinct steps^27^ and in the final step, we used the k-mesh density of 0.05 in units of 2*π* Å^*−* 1^. The force convergence criterion for structure relaxation was kept 0.02 eV/ Å. For next step structure generation, some of them were randomly selected from a pool of planar group. The remaining potential structures were obtained through diverse transmutations operations on lowest energy structures from the last step. Furthermore, we repeated these steps until the same lowest energy structure was achieved in each step at least 4-5 times. The lowest energy structures by running the single and two pairs per unit cell are used for the further calculations. To avoid any chance of trapping into local minima, we performed the double-checking at each universal structure relaxation. For SIESTA calculations, we used the Troullier–Martins norm-conserving pseudopotential for the treatment of core electrons. Variationally optimized double-ζ plus polarized basis sets were used to simulate the valence electron^28,29^. The generalized gradient approximation by applying the Perdew−Burke−Ernzerhof (PBE) exchange-correlation functional was used^30^. The SIESTA code was also used to perform molecular dynamics (MD) calculations using NTV ensemble and the smallest time step was taken as 1.0 fs^31^. All magnetic ground states calculations were performed using the spin-polarized density functional theory as implemented in Vienna ab-initio simulation package (VASP)^32,33^ with the projector augmented wave (PAW)^34^. We employed the QUANTUM-ESPRESSO (QE) package^35^ to calculate the phonon dispersions and electron-phonon coupling. To keep the consistency, the PAW pseudopotential was employed to model the boron and manganese ions. For the phonon calculations, the structures were fully relaxed until the Hellman-Feynman force on each atom was less than 10^5^ Ry/Bohr. We analyzed the physical properties of the electron-phonon coupling (EPC) using the Migdal^36^ and Eliashberg theory^37^. For the momentum and frequency integrated total electron-phonon coupling (EPC) parameter λ, the k-mesh, q-grids and Gaussian smearing parameter are chosen after ensuring the convergence.

## References

[1] Miró P, Audiffred M, Heine T (2014). An atlas of two-dimensional materials. Chem. Soc. Rev..

[2] Liu H (2014). Phosphorene: An Unexplored 2D Semiconductor with a High Hole Mobility. ACS Nano.

[3] Heising J, Kanatzidis MG (1999). Exfoliated and Restacked MoS_2_ and WS_2_:  Ionic or Neutral Species? Encapsulation and Ordering of Hard Electropositive Cations. J. Am. Chem. Soc..

[4] Mahmood J (2015). Nitrogenated holey two-dimensional structures. Nat. Commun..

[5] Hashmi A, Farooq MU, Hu T, Hong J (2015). Spin-Dependent Transport and Optical Properties of Transparent Half-Metallic g-C4N3 Films. J. Phys. Chem. C.

[6] Algara-Siller G (2014). Triazine-Based Graphitic Carbon Nitride: a Two-Dimensional Semiconductor. Angew. Chem. Int. Ed..

[7] Hashmi A, Farooq MU, Khan I, Son J, Hong J (2017). Ultra-high capacity hydrogen storage in a Li decorated two-dimensional C_2_N layer. J. Mater. Chem. A.

[8] Sun Z (2014). Generalized self-assembly of scalable two-dimensional transition metal oxide nanosheets. Nat. Commun..

[9] Zhang W (2013). Unexpected Stable Stoichiometries of Sodium Chlorides. Science.

[10] Mannix AJ (2015). Synthesis of borophenes: Anisotropic, two-dimensional boron polymorphs. Science.

[11] Wu X (2012). Two-Dimensional Boron Monolayer Sheets. ACS Nano.

[12] Zheng F, Wang Z, Kang W, Zhang P (2013). Antiferromagnetic FeSe monolayer on SrTiO_3_: The charge doping and electric field effects. Sci. Rep..

[13] Yelland EA (2005). Superconductivity induced by spark erosion in ZrZn_2_. Phys. Rev. B.

[14] Penev ES, Kutana A, Yakobson BI (2016). Can Two-Dimensional Boron Superconduct?. Nano Lett..

[15] Zhao Y, Zeng S, Ni J (2016). Phonon-mediated superconductivity in borophenes. Appl. Phys. Lett..

[16] Shao DF, Lu WJ, Lv HY, Sun YP (2014). Electron-doped phosphorene: A potential monolayer superconductor. EPL Europhys. Lett..

[17] Zhang Y (2016). Superconducting Gap Anisotropy in Monolayer FeSe Thin Film. Phys. Rev. Lett..

[18] Zhu X (2016). Signature of coexistence of superconductivity and ferromagnetism in two-dimensional NbSe_2_ triggered by surface molecular adsorption. Nat. Commun..

[19] Ma, S. *et al*. Manganese mono-boride, an inexpensive room temperature ferromagnetic hard material. *Sci. Rep*. **7**, (2017). 10.1038/srep43759 PMC5338324 28262805

[20] Tan D (2017). Anisotropic optical and electronic properties of two-dimensional layered germanium sulfide. Nano Res..

[21] Farooq MU, Hashmi A, Hong J (2015). Anisotropic bias dependent transport property of defective phosphorene layer. Sci. Rep..

[22] Yang L-M, Frauenheim T, Ganz E (2015). The new dimension of silver. Phys. Chem. Chem. Phys..

[23] Yang L-M, Dornfeld M, Frauenheim T, Ganz E (2015). Glitter in a 2D monolayer. Phys. Chem. Chem. Phys..

[24] Kohn W (1959). Image of the Fermi Surface in the Vibration Spectrum of a Metal. Phys. Rev. Lett..

[25] Glass CW, Oganov AR, Hansen N (2006). USPEX—Evolutionary crystal structure prediction. Comput. Phys. Commun..

[26] Oganov AR, Glass CW (2006). Crystal structure prediction using ab initio evolutionary techniques: Principles and applications. J. Chem. Phys..

[27] Soler JM (2002). The SIESTA method for ab initio order-N materials simulation. J. Phys. Condens. Matter.

[28] Anglada E, Soler M, Junquera J, Artacho E (2002). Systematic generation of finite-range atomic basis sets for linear-scaling calculations. Phys. Rev. B.

[29] Junquera J, Paz Ó, Sánchez-Portal D, Artacho E (2001). Numerical atomic orbitals for linear-scaling calculations. Phys. Rev. B.

[30] Büttiker M, Imry Y, Landauer R, Pinhas S (1985). Generalized many-channel conductance formula with application to small rings. Phys. Rev. B.

[31] Anglada E, Junquera J, Soler JM (2003). Efficient mixed-force first-principles molecular dynamics. Phys. Rev. E.

[32] Kresse G, Hafner J (1994). Ab initio molecular-dynamics simulation of the liquid-metal-amorphous-semiconductor transition in germanium. Phys. Rev. B.

[33] Kresse G, Furthmüller J (1996). Efficient iterative schemes for ab initio total-energy calculations using a plane-wave basis set. Phys. Rev. B.

[34] Blöchl PE (1994). Projector augmented-wave method. Phys. Rev. B.

[35] Giannozzi P (2009). Quantum Espresso: a modular and open-source software project for quantum simulations of materials. J. Phys. Condens. Matter.

[36] Migdal AB (1958). Interaction Between Electrons and Lattice Vibrations in a Normal Metal. Sov. Phys. JETP.

[37] Eliashberg GMNteractions (1960). Between Electrons and Lattice Vibrations in a Superconductor. Sov. Phys. JETP.

